# Rapidly Dissolving Microneedles Incorporating Lidocaine Hydrochloride: A PVP/PVA-Based Approach for Local Anesthesia

**DOI:** 10.3390/pharmaceutics17091100

**Published:** 2025-08-23

**Authors:** Su Young Jin, Eugene Jae-Jin Park, Sae Min Kwon, Hyoung-Seok Jung, Dong Wuk Kim

**Affiliations:** 1 BK21 FOUR Community-Based Intelligent Novel Drug Discovery Education Unit, Vessel-Organ Interaction Research Center (VOICE, MRC), College of Pharmacy and Research Institute of Pharmaceutical Sciences, Kyungpook National University, Daegu 41566, Republic of Korea; jin0123kr@naver.com; 2 Spine Center, Bogang Hospital, Daegu 42801, Republic of Korea; 3 Department of Neurosurgery, Dongsan Medical Center, Keimyung University School of Medicine, Daegu 42601, Republic of Korea; 4 Hand and Upper Extremity Surgery, Department of Orthopaedic Surgery, Chung-Ang University Gwangmyeong Hospital, Gwangmyeong 14353, Republic of Korea

**Keywords:** lidocaine hydrochloride, microneedles, local anesthetic, polyvinylpyrrolidone, polyvinyl alcohol, transdermal drug delivery

## Abstract

**Background/Objectives:** Lidocaine is a widely used local anesthetic, but injections and topical creams are often painful or slow in onset. This study aimed to develop dissolving microneedles incorporating lidocaine hydrochloride for rapid and convenient local anesthesia. **Methods:** Six formulations were prepared with polyvinylpyrrolidone (PVP) and polyvinyl alcohol (PVA) and evaluated for mechanical strength, skin insertion, drug release, and transdermal permeability. **Results:** Sharp pyramidal microneedles were successfully fabricated, with PVP–PVA mixtures producing stronger needles than single polymers. The optimized F5 formulation showed high strength (>32 N), efficient skin insertion (four parafilm layers), and rapid release (>80% within 15 min). In ex vivo studies, F5 delivered >600 µg/mL lidocaine in 15 min, over three times the therapeutic level and much faster than Emla cream (5%). **Conclusions:** PVP–PVA microneedles represent a promising platform for painless, rapid local anesthesia, combining the benefits of injections and topical creams while minimizing their drawbacks.

## 1. Introduction

In modern clinical practice, procedures such as venipuncture, dental treatments, and skin biopsies are among the primary causes of pain and often provoke psychological distress in patients [1,2,3]. Even brief medical interventions can trigger anticipatory anxiety, elevating stress levels and reducing treatment adherence. Although topical anesthetics are widely used to alleviate discomfort, most fail to effectively penetrate biological barriers, such as the skin or mucosal tissues [4].

The stratum corneum of the skin serves as a major physical barrier to passive drug diffusion, often delaying the onset of local anesthetic effects or preventing the achievement of adequate drug concentrations. Consequently, injection-based methods remain widely used to induce anesthesia rapidly. Hypodermic needle injections have been employed for over a century as a common alternative to oral drug delivery. However, subcutaneous injections are invasive and may cause pain, increase the risk of infection, and induce needle phobia, factors that paradoxically contribute to additional patient discomfort. They also generate sharp, biologically hazardous medical waste and require administration by trained medical personnel. Furthermore, in vaccine administration, hypodermic needles can penetrate muscle tissue, which elicits a weaker immune response than the skin. Against this background, demand for more effective, noninvasive local drug delivery technologies is steadily increasing [5,6].

**Figure 1:**
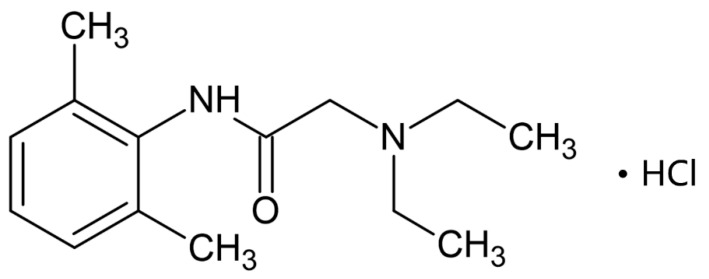
Structure of lidocaine hydrochloride.

Lidocaine is one of the most extensively used local anesthetics to address this clinical need [7,8]. As an amide-type anesthetic, it is also employed in the management of neuropathic pain and the treatment of ventricular arrhythmia. As shown in Figure 1, lidocaine comprises three components: a hydrophilic amine group, an aromatic residue, and an intermediate chain linking the two [9]. As a local anesthetic, lidocaine acts by reversibly blocking voltage-gated sodium channels, thereby inhibiting nerve impulse propagation [10]. It offers several pharmacological advantages, including rapid onset, appropriate duration of action, and low systemic toxicity. However, conventional topical formulations, such as creams and gels, exhibit poor skin penetration, leading to delayed therapeutic effects, a notable limitation in clinical contexts requiring rapid anesthesia.

To address these limitations, microneedle (MN)-based drug delivery systems have emerged as a promising alternative. MNs are an innovative technology capable of delivering drugs directly into the skin or systemic circulation without causing pain. These needles are arranged in arrays, are extremely small, and are less than 1000 mm in length. Unlike conventional injection needles, MNs create microscopic pathways that enable painless drug administration [11,12]. Dissolving microneedles (DMNs), produced by directly incorporating lidocaine into biodegradable polymers, represent a novel delivery platform. These MNs physically penetrate the skin barrier and dissolve upon contact with interstitial fluid, rapidly releasing the drug into the target tissue. Compared with conventional topical formulations, DMNs markedly improve drug delivery efficiency while leaving no solid needle component in the tissue, thereby eliminating the need for post-application removal. Additionally, they reduce the generation of biohazardous waste by using biodegradable materials. In contrast, coated MNs have limited drug loading capacity, poor coating uniformity, and suboptimal biocompatibility, which restrict their applicability in broad clinical settings. In this context, DMNs are increasingly recognized as a promising alternative.

Several studies have already explored lidocaine-loaded DMNs using polymers such as hyaluronic acid, demonstrating rapid onset and prolonged anesthetic effects [13]. However, these approaches often required complex materials or fabrication processes, which may limit reproducibility and scalability [14]. In contrast, our work focused on a simple and reproducible fabrication strategy, systematically optimized to achieve robust mechanical strength, efficient skin penetration, and rapid lidocaine release. The optimized system delivered therapeutic concentrations within 15 min, significantly faster than Emla cream, thereby highlighting the novelty and clinical practicality of our approach.

In this study, we developed a DMN system for noninvasive drug delivery to effectively overcome the skin barrier and achieve the rapid local anesthetic action of lidocaine. We selected polyvinylpyrrolidone (PVP) and polyvinyl alcohol (PVA) as base materials owing to their excellent biocompatibility and favorable mechanical properties. Various formulation ratios were prepared and comprehensively evaluated for drug delivery efficiency, mechanical stability, and skin insertion performance. This study aimed to provide a practical and safer alternative to conventional topical anesthetic formulations and to advance the development of MN-based local anesthetic systems.

## 2. Materials and Methods

### 2.1. Materials

Lidocaine hydrochloride (Figure 1) was sourced from Alfa Aesar (Tewksbury, MA, USA). PVP K30 was obtained from Sigma-Aldrich (Gillingham, UK), and PVA was provided by Hanmi Pharm. Co., Ltd. (Suwon, Republic of Korea). Porcine skin (Micropig^®^ Franz membrane; 2 × 2 cm × 400 μm) was sourced from Medi Kinetics Co., Ltd. (Pyeongtaek, Republic of Korea). All reagents were of analytical grade and used without further purification.

### 2.2. Fabrication of Lidocaine Hydrochloride DMNs

The MN formulations were prepared using a double-casting technique. The molds had a pyramidal needle shape (Micropoint Technologies, Singapore, Singapore) with a density of 10 × 10, a height of 700 μm, a base width of 200 μm, and a pitch of 500 μm. Each mold was filled with 50 mg of the lidocaine-containing formulation, followed by vacuum degassing for 30 min and centrifugation at 4000 rpm for 30 min to ensure complete filling of the mold cavities. Excess formulation on the mold surface was removed, and the molds were dried at room temperature for 1 h to complete the first casting step. In the second casting step, 100 mg of a lidocaine-free formulation was applied as the support layer for the MNs. The molds were maintained at 37 °C for 24 h and then dried for an additional 24 h at room temperature. Finally, the dried MNs were manually removed from the molds and stored in sealed containers until use [15].

### 2.3. Morphological Characterization

The shape and size of the lidocaine-containing MNs were examined using a stereomicroscope (SMZ18, Nikon, Tokyo, Japan). In addition, the morphological characteristics and drug crystallinity of pure LDH, the physical mixture (PM), the MN array, and the MN tips were analyzed using field emission scanning electron microscopy (FE-SEM, SU8230, Hitachi, Tokyo, Japan) at an accelerating voltage of 5.0 kV. Before analysis, all samples were mounted on brass specimen holders with double-sided adhesive tape and sputter-coated with platinum at a deposition rate of 6 nm/min for 4 min under a vacuum of 0.8 Pa and a current of 15 mA, using an EmiTech Sputter Coater K575K (Quorum Technologies Ltd., Lewes, UK) to enhance conductivity.

### 2.4. Mechanical Testing of MNs

#### 2.4.1. Mechanical Strength Test

MNs must possess sufficient mechanical strength to effectively penetrate the skin. To assess the mechanical strength and insertion characteristics of the MNs, compression tests were conducted using a texture analyzer (TA, CT3-4500, Brookfield, Middleboro, MA, USA) in compression mode. The MNs were affixed to a TA4/100 cylindrical probe with the needle tips aligned to the probe axis using double-sided adhesive tape. After contact with the support housing, a trigger force of 0.49 N was applied, and the TA began data collection. The probe was then lowered vertically at a constant speed of 1 mm/s until a force of 32 N was reached. This force represents the maximum average force that an individual can apply when pressing an MN against the skin [15]. The initial height (*H*1) and post-compression height (*H*2) of LDH-DMN were measured using a stereomicroscope (SMZ18). The percentage reduction in LDH-DMN height after compression force was calculated using the following equation [16]:

$$Reduction\;height\;\left(\%\right)=\;\frac{\left[H1-H2\right]}{H1}\times 100\%$$

#### 2.4.2. Penetration Ability Test

The skin insertion efficacy of MNs was evaluated using Parafilm M^®^ (Amcor, Neenah, WI, USA) as a skin simulant [17]. A skin model approximately 1 mm thick was prepared by stacking eight layers of Parafilm M sheets. The MNs were inserted into the prepared Parafilm model using a TA. After insertion, the MNs were removed, and the pores formed in each Parafilm layer were quantified using a stereomicroscope (SMZ18) [18].

$$Pentration\;of\;n\;layer=\frac{number\;of\;holes\;in\;n\;layer}{total\;number\;of\;holes}\times 100\%$$

#### 2.4.3. Skin Insertion Test

To evaluate the skin penetration performance of the MNs, a depilated mouse skin model, considered similar to human skin, was used. The MNs were inserted into the skin by applying thumb pressure for 30 s. After compression, insertion was visually observed. The mouse skin was stored at −20 °C until use. Before MN insertion, the skin was equilibrated in phosphate-buffered saline (PBS; pH 7.4) for 30 min, and excess surface moisture was removed. After insertion, the skin was stained with a 1% methylene blue solution for 5 min. The resulting pores on the skin surface were observed under a stereomicroscope [19,20]. The penetration performance of the MNs was evaluated based on the successful formation of visible perforations in the mouse skin. In addition, the skin penetration ability of the MNs was evaluated using Optical Coherence Tomography (OCT) at Kyungpook National University (Daegu, Republic of Korea). Rat skin was used, and cross-sectional images were obtained before and after MN insertion to quantitatively measure the maximum penetration depth from the skin surface.

### 2.5. Physicochemical Characteristics Study of LDH-DMNs

#### 2.5.1. Differential Scanning Calorimetry (DSC)

The thermal properties of pure LDH, PVP K-30, PVA, the PM, and the optimized formulation were evaluated using a differential scanning calorimeter (DSC, Q20; TA Instruments, Newcastle, DE, USA). Each sample was hermetically sealed in an aluminum pan and heated from 30 to 230 °C at a rate of 10 °C/min under a nitrogen gas flow of 50 mL/min. Calorimetric data were collected and analyzed using the TA Universal Analysis 2000 software.

#### 2.5.2. Powder X-Ray Diffraction (PXRD)

The crystalline structures of pure LDH, PVP K-30, PVA, the PM, and the optimized formulation were analyzed using a powder X-ray diffractometer (D/MAX-2500; Rigaku; Tokyo, Japan) equipped with Cu Kα radiation (λ = 1.54178 Å, 40 kV, and 40 mA). Diffraction patterns were recorded at ambient temperature over a 2θ range of 2° to 60°, with a step size of 0.05°/s.

#### 2.5.3. Fourier Transform Infrared (FTIR) Spectroscopy

FTIR spectroscopy was performed to obtain infrared spectra (400–4000 cm^−1^) for pure LDH, PVP K-30, PVA, the PM, and the optimized formulation. Spectral data were collected using a PerkinElmer Frontier FTIR spectrometer (PerkinElmer, Inc., Waltham, MA, USA). For the analysis, the optimized MN samples were prepared by isolating the needle layer.

### 2.6. High-Performance Liquid Chromatography (HPLC) Analysis

HPLC analysis was performed using a Dionex UltiMate 3000 (Thermo Fisher Scientific, Bremen, Germany) equipped with a reversed-phase C18 column (Hypersil™ ODS-2, 5 μm, 4.6 mm id × 150 mm; Thermo Fisher Scientific, Waltham, MA, USA) and an ultraviolet detector. The mobile phase was prepared by dissolving 50 mL of glacial acetic acid in 930 mL of distilled water, adjusting the pH to 3.4 with 0.1 N sodium hydroxide, and mixing the buffer with acetonitrile at a 70:30 (*v*/*v*) ratio. The column temperature was maintained at 25 °C, the detection wavelength was set to 254 nm, the injection volume was 20 μL, and the flow rate was 1.0 mL/min.

### 2.7. Drug Content Determination

For drug content determination, accurately weighed MN samples were dissolved in 50 mL of PBS (pH 7.4). The solution was filtered through a 0.22 μm syringe filter and analyzed using the HPLC method described in Section 2.6. Drug content was quantified against a standard calibration curve of lidocaine hydrochloride. All measurements were performed in triplicate and expressed as mean ± standard deviation (SD).

### 2.8. In Vitro Release Study

To evaluate the drug release profile of MNs, the dissolution medium was prepared by adding 100 mL of PBS (pH 7.4) to a 250 mL glass beaker and maintaining the temperature at 32 ± 0.5 °C. Release studies were performed by placing the MNs in sinker baskets [21]. The MNs were completely immersed in the dissolution medium, and 1 mL aliquots were withdrawn at predetermined time points (5, 10, 15, 20, 30, 45, and 60 min). Each withdrawn volume was immediately replaced with an equal volume of fresh PBS (pH 7.4). The collected samples were analyzed using HPLC (Thermo Fisher Scientific, Bremen, Germany) at a detection wavelength of 254 nm. All experiments were performed in triplicate, and results were reported as mean ± SD.

### 2.9. Ex Vivo Skin Permeation Study

To evaluate the percutaneous penetration efficacy of MNs, experiments were conducted using Franz diffusion cells within a percutaneous diffusion cell system (DHC-6TD, LOGAN Instruments, Somerset, NJ, USA) to measure lidocaine release. The Franz diffusion cell, a standard device for skin permeation studies, was employed with pig skin (Micropig^®^ Franz membrane; APURES, Pyeongtaek, Republic of Korea) obtained from Medikinetics (Pyeongtaek, Republic of Korea). The skin was equilibrated in PBS (pH 7.4) at 32 ± 0.5 °C for 30 min and then mounted between the two compartments of the Franz diffusion cell, with the stratum corneum facing the donor compartment. As a control, EMLA cream [22], which contains 25 mg of lidocaine and 25 mg of prilocaine per g and is widely used in clinical practice, was included. Formulations F5 and F6, each containing approximately 2.5 mg of lidocaine, served as the experimental groups. F3 was excluded due to insufficient mechanical strength, and F4 was excluded because of its slower drug release compared with F5; thus, only F5 (optimized formulation) and F6 (control) were selected for the ex vivo permeation study. The receptor compartment was filled with 5 mL of freshly prepared PBS (pH 7.4). The system was maintained at 32 ± 0.5 °C with continuous stirring at 600 rpm, and the effective diffusion area was 1.77 cm^2^. At predetermined intervals (15, 30, 45, and 60 min), 1 mL aliquots were withdrawn from the receptor compartment and replaced with an equal volume of fresh PBS. The collected samples were analyzed using HPLC (Thermo Fisher Scientific, Bremen, Germany). Cumulative drug permeation (*Qn*) was calculated using the standard equation.

$$Qn=\;\sum_{i=1}^{n}\left(C_{i}\times V\right)$$

### 2.10. Statistical Analysis

Statistical analyses were conducted using Minitab^®^ software (version 21; Minitab Inc., State College, PA, USA). Differences between the two groups were assessed using the Student’s *t*-test. A *p*-value of <0.05 was considered statistically significant. All data are presented as mean ± SD.

## 3. Results and Discussion

### 3.1. MN Manufacturing Process

In this study, the lidocaine concentration was fixed at 5% *w*/*w*, consistent with the commercial reference product (Emla^®^ cream, 5%; Mitsubishi Tanabe Pharma Korea, Seoul, Republic of Korea) and previously reported lidocaine-loaded MN formulations, thereby ensuring both clinical relevance and comparability.

When the PVP content was reduced below 20%, patch breakage increased markedly during drug release, and the mechanical strength of the MNs fell below the required skin penetration threshold of 32 N. In contrast, increasing the PVP content to 30% improved solution flow and enabled complete filling of the mold cavity. However, this reduced hardness limits its suitability for independent use.

Using PVA alone presented additional manufacturing challenges. At high concentrations, PVA hindered effective centrifugation, resulting in uneven MN formation, while very low concentrations produced insufficient hardness [23]. To address these issues, incorporating 5–20% PVA reinforced the PVP matrix, significantly enhancing MN strength beyond the 32 N threshold.

**Figure 2:**
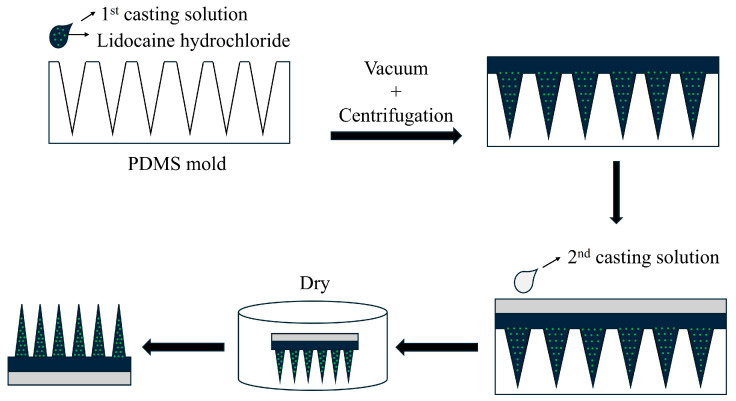
Preparation process of lidocaine–PVA/PVP MNs.

Based on compositional analysis, the formulation listed in Table 1 was selected for further evaluation. The manufacturing process involved layering the solution into the mold, centrifuging at 4000 rpm for 30 min to ensure complete filling, applying a drug-free mixture as a support layer, and drying the structure in the mold (Figure 2). Shorter centrifugation times led to incomplete filling, deformation of MN tips, and reduced mechanical strength. Thus, optimal shape and consistent manufacturing quality were achieved by maintaining centrifugation at 4000 rpm for 30 min.

### 3.2. Characterization of MNs

**Figure 3:**
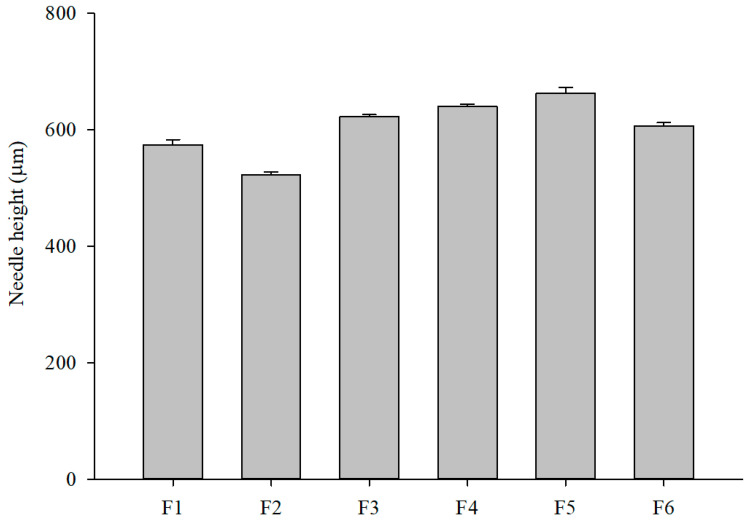
Effect of PVP–PVA polymer ratio on MN height (*n* = 3).

Successful fabrication of MNs using the PDMS mold and centrifugal filling method was confirmed via stereomicroscopy. Each mold contained a 10 × 10 array of MNs, covering approximately 10 × 10 mm^2^. The MNs accurately replicated the mold geometry, measuring approximately 800 μm in height, 200 μm at the base, and separated by a 500 μm gap. Pure formulations F1 (PVP only) and F2 (PVA only) produced shorter needles than PVP–PVA blend formulations. Within the blends, needle length increased as the PVP concentration increased; however, formulation F6, which had a higher PVA content, exhibited relatively shorter needles (Figure 3). These differences in needle length are mainly attributed to variations in solution viscosity affecting mold filling, as higher PVP content improved flow while higher PVA content increased viscosity and limited penetration. In addition, the final microneedle length (~600 μm) was shorter than the 800 μm mold depth due to polymer shrinkage during drying, a phenomenon commonly observed in DMN fabrication.

**Figure 4:**
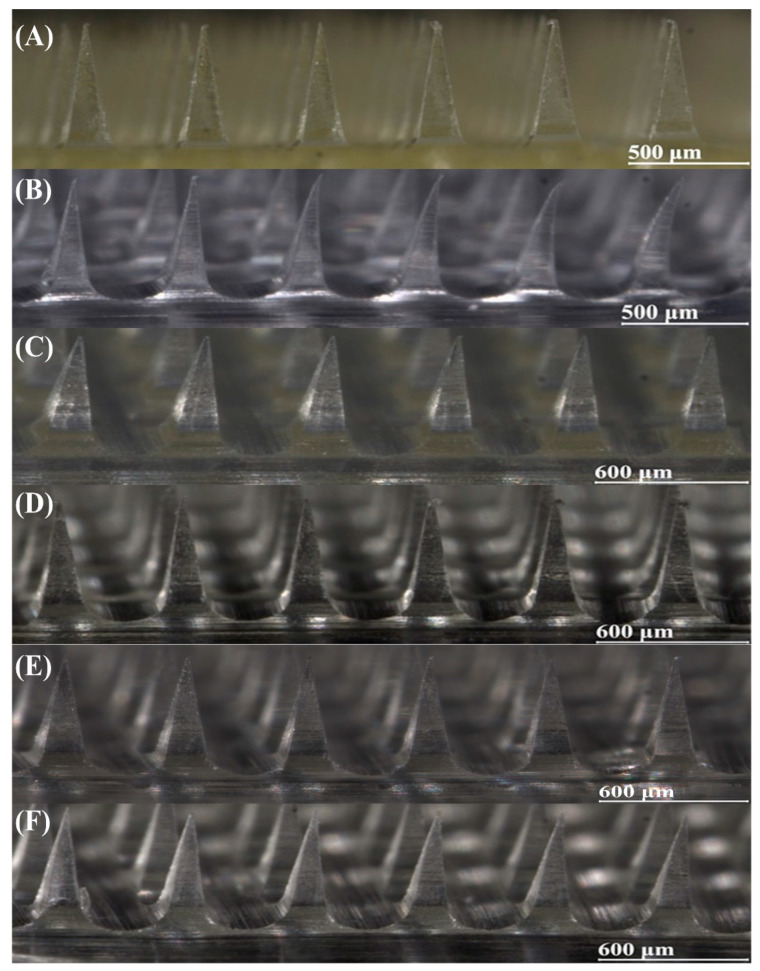
MN morphology of formulations. (**A**) F1, (**B**) F2, (**C**) F3, (**D**) F4, (**E**) F5, and (**F**) F6.

MNs must maintain a complete structure capable of penetrating the stratum corneum. Optical microscopy (Figure 4) revealed that all six formulations consistently produced well-formed needles matching the mold design, demonstrating effective demolding with intact morphology. Formulation F1 appeared pale yellow due to polymer characteristics, whereas formulation F2 exhibited needle bending caused by high fluidity. All formulations showed uniform structures without bubbles or clumps, indicating excellent quality. Although a slight needle length reduction occurred during solvent evaporation [23], this decrease may facilitate painless application by effectively penetrating the approximately 10 μm thick stratum corneum without stimulating dermal vessels [15].

**Figure 5:**
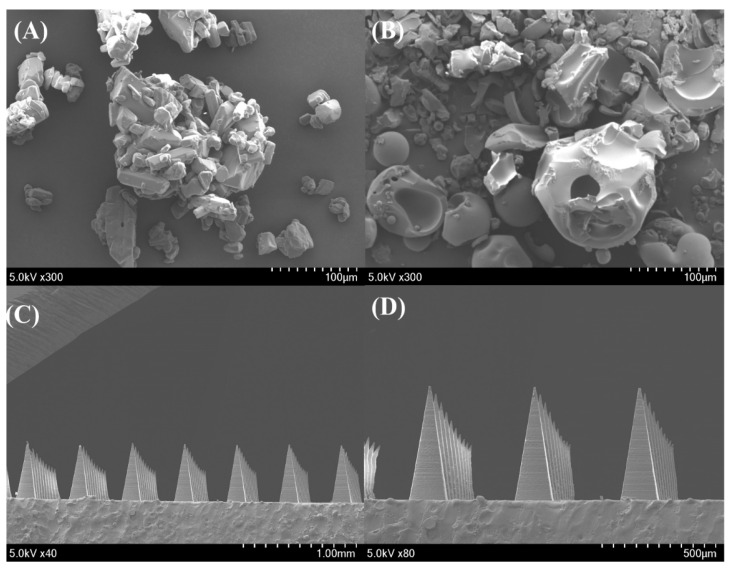
SEM images of (**A**) lidocaine hydrochloride (×300), (**B**) PM (×300), (**C**) MN array (×40), and (**D**) MN tips (×80).

Surface morphology and crystallinity of the pure drug, PM, and MN formulations were examined via SEM (Figure 5). The pure drug exhibited characteristic crystalline structures, whereas the PM showed a heterogeneous surface with visible drug particles dispersed within the polymer matrix. In contrast, the MN surfaces appeared smooth and uniform, with no visible crystalline residues, indicating that the drug was well incorporated in an amorphous or molecularly dispersed state within the polymer network.

### 3.3. Mechanical Properties of MNs

#### 3.3.1. Mechanical Strength Results

MNs are essential components of transdermal drug delivery systems, enabling efficient drug administration by creating microchannels in the skin. To ensure successful performance, MNs must possess sufficient mechanical strength to withstand both the force applied during skin insertion and external stresses that may occur during storage, transport, or handling [24]. Adequate strength prevents breakage or deformation, ensuring safe application and effective penetration of the stratum corneum.

Previous studies have shown that MNs composed solely of PVP exhibit limited mechanical strength, reducing their ability to penetrate the skin effectively [25]. To address this limitation, blending PVP with PVA has been proposed as an effective strategy to enhance mechanical properties. This improvement is attributed to hydrogen bonding between the hydroxyl groups (–OH) of PVA and the carbonyl groups (–C=O) of PVP, which strengthens the polymer network [26]. The PVA/PVP blend enhances the mechanical strength of MN tips and provides a stable structure that supports consistent skin insertion. Consequently, this combination is commonly used in MN fabrication. Moreover, reports have demonstrated that MNs composed of both PVP and PVA exhibit superior insertion performance and retain structural height more effectively after application compared with MNs made from either polymer alone [27]. Based on these findings, this study aimed to improve MN mechanical strength using PVA–PVP blend formulations.

**Figure 6:**
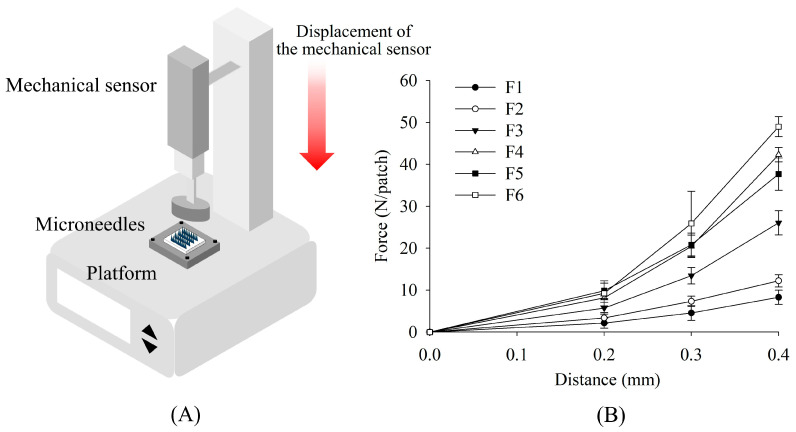
(**A**) Experimental setup illustration. (**B**) Effect of polymer ratio on mechanical strength (mean ± SD, *n* = 3).

This study evaluated the mechanical strength of MNs prepared with different polymer concentrations under compression (Figure 6). MNs were mounted on a support, and a gradually increasing force was applied using a TA. Force and displacement were continuously recorded to generate force–displacement curves, with the measured forces normalized to represent the force applied per single MN. Results indicated that MN strength increased with total polymer concentration across all formulations. Deformation curves displayed a consistent trend, with applied force increasing steadily as the needles were compressed. Among the PVP–PVA blends, formulation F6—containing a higher PVA content—exhibited the highest mechanical strength (Table 2). Lidocaine-loaded MNs in this group reached a maximum force of 0.32 N per needle at a displacement of 0.4 mm, substantially exceeding the minimum insertion force required to penetrate the skin (approximately 0.098 N per needle) [28], indicating that the developed MNs possess sufficient mechanical strength for reliable skin insertion.

#### 3.3.2. Penetration Ability Results

**Figure 7:**
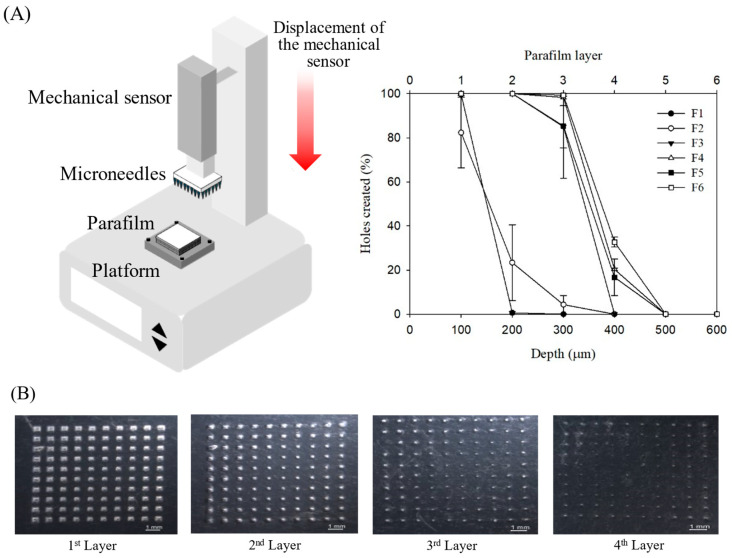
(**A**) Graph of penetration percentage of the parafilm layer. (**B**) Parafilm layers post-MN insertion.

The skin insertion efficacy of MNs with varying needle lengths was investigated in this study. Mechanical strength decreased significantly with increasing PVP concentration, likely due to PVP dissolution in the aqueous environment during skin insertion, which weakens the MN structure. To enhance mechanical strength, a composite material of PVA and PVP was employed. This combination is widely used in MN fabrication, providing an optimal structure that improves mechanical strength and skin penetration. Previous studies have demonstrated that PVA/PVP mixtures exhibit superior insertion performance and reduced needle height loss compared with MNs composed solely of PVP or PVA. The insertion force of MN is a key determinant of skin permeability and drug delivery efficiency. Parafilm^®^ has been established as a reliable artificial membrane for simulating skin [16], with a single layer approximately 126 μm thick; the number of penetrated layers corresponds to insertion depth. Test results demonstrated that all formulations completely (100%) penetrated the first Parafilm^®^ layer. Formulations F3, F4, and F5 achieved excellent penetration depth, reaching the fourth Parafilm^®^ M layer (Figure 7). These findings indicate that the prepared MNs can effectively penetrate the stratum corneum and epidermis, bypassing the depth of skin pain receptors. These results are consistent with previous reports showing that MN arrays of similar strength, which typically penetrate 2–3 layers of Parafilm^®^ M, successfully penetrate animal skin in vitro and in vivo [24].

#### 3.3.3. Skin Insertion Results

**Figure 8:**
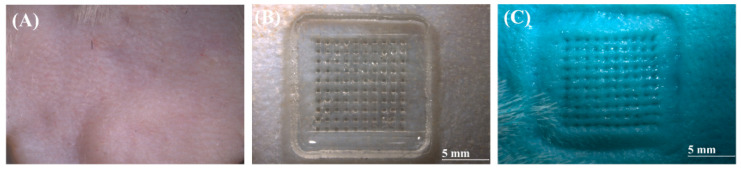
Ex vivo insertion test. (**A**) Optical micrograph of a typical rat skin sample. (**B**) MNs after insertion in rat skin. (**C**) Stained rat skin following MN treatment.

The skin penetration efficacy of the MNs was evaluated using depilated rat skin. MN patches were applied with thumb pressure for 30 s. After removal, a 1% methylene blue solution was applied for 5 min to stain the insertion sites [29]. Excess dye was removed with ethanol, and the skin surface was thoroughly cleaned. Examination of the stained skin revealed clear perforation marks for all MNs, indicating 100% penetration (Figure 8). These results confirm that the fabricated MNs were successfully inserted into rat skin and effectively created microchannels.

**Figure 9:**
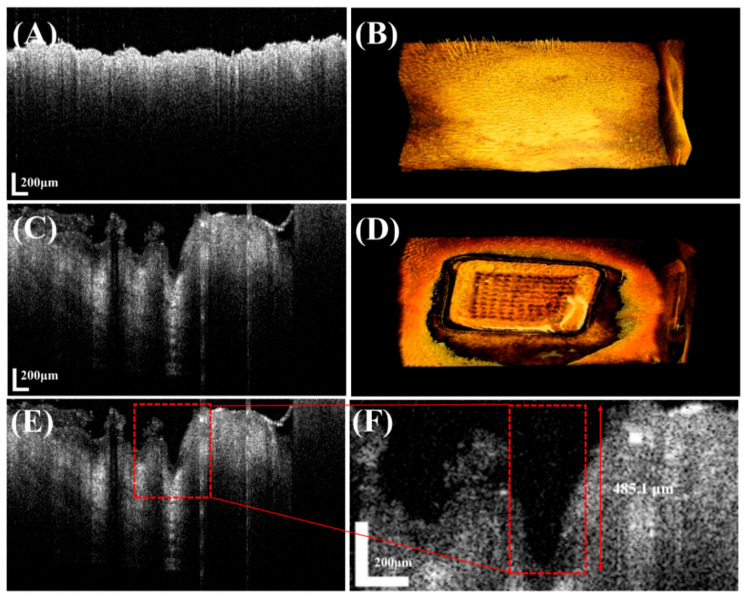
Rat skin penetration depth analysis based on 2D (**A**,**C**,**E**,**F**) and 3D (**B**,**D**) OCT images before and after MN insertion. (**A**,**B**) Before MN insertion, (**C**,**D**) after MN insertion, (**E**) detailed OCT image before skin penetration, and (**F**) more magnified detailed OCT image after skin penetration.

To evaluate the skin penetration depth of the MN devices, they were inserted into excised rat skin, and tissue changes were analyzed using OCT. Figure 9A shows an OCT image before insertion, clearly depicting the stratum corneum and underlying skin structures, while Figure 9C shows an image after insertion, visually confirming successful penetration. Measurements indicated that the MNs penetrated to a depth exceeding 50% of their initial needle length, reaching the dermis beyond the stratum corneum, consistent with previously reported insertion depths [30]. These results suggest that the MN devices can effectively deliver lidocaine into the skin in vivo, penetrate the stratum corneum, and reach the dermis, thereby enhancing local anesthetic effects or contributing to other therapeutic outcomes. By quantitatively measuring and visually confirming skin penetration depth using OCT, we demonstrated that the developed MNs successfully overcome the stratum corneum barrier and reach the dermis, highlighting their potential as a transdermal drug delivery system for various future applications [31].

### 3.4. Physicochemical Characteristics of LDH-DMNs

#### 3.4.1. DSC

**Figure 10:**
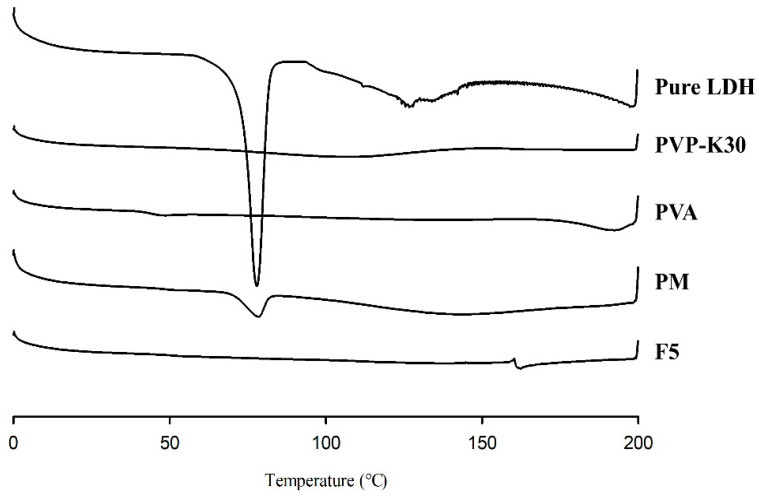
DSC thermograms.

As shown in Figure 10, lidocaine exhibited a distinct and prominent endothermic peak at 77.8 °C, corresponding to its melting point [32]. In the PM, however, the intensity of this endothermic transition was markedly reduced, likely due to the relatively low concentration of lidocaine compared with the PVP-K30 and PVA components. Additionally, this peak was absent in the DSC profile of the optimized aqueous formulation, which can be attributed to the dissolution of lidocaine hydrochloride within the PVA/PVP matrix and aqueous medium.

#### 3.4.2. PXRD

**Figure 11:**
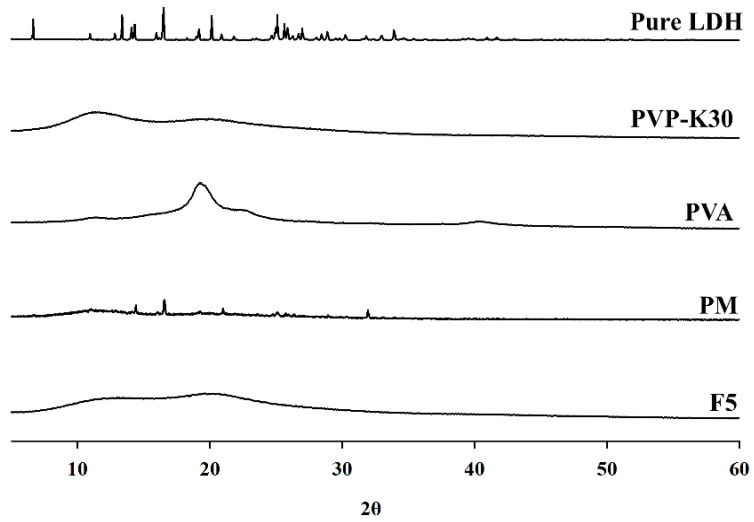
PXRD patterns.

XRD is a non-destructive analytical technique used to investigate the crystal structure of materials and is particularly useful for characterizing polymer composite formation. The results (Figure 11) showed broad diffraction peaks at 2θ = 19.2° and 40.1°, with the prominent peak at 19.2° corresponding to reflections from aligned polymer chains within the crystalline domains. PVP powder exhibited two broad diffraction halos at 2θ = 11.6° and 19.6°, indicating amorphous characteristics and confirming the absence of crystalline structure. In contrast, XRD analysis confirmed that PVA possessed a crystalline structure, while PVP remained amorphous [32,33]. Adding PVP to the blend reduced PVA crystallinity, suggesting that interactions between PVA and PVP partially disrupted the crystalline structure of PVA, thereby promoting the formation of amorphous domains. These findings are consistent with complementary analytical results, indicating that polymer interactions alter the structural properties of the composites [32]. XRD analysis of lidocaine HCl revealed several sharp peaks, confirming its high crystallinity. In contrast, no crystalline pattern corresponding to lidocaine HCl was detected in the PM or F5 samples, as evidenced by the absence of drug-related peaks and the transparency of the matrix. These results indicate that lidocaine HCl was uniformly dispersed at the molecular level within the polymer matrix.

#### 3.4.3. FTIR

The ATR-FTIR spectrum of lidocaine HCl exhibited characteristic peaks at 3384 cm^−1^ (N–H stretching), 2946 cm^−1^ (C–H stretching), 1654 cm^−1^ (amide I band, C=O stretching), and 1474 cm^−1^ (amide II band, C–N stretching). These spectral features confirm the molecular structure of lidocaine HCl while indicating the absence of chemical interaction with PVA or PVP, suggesting that it exists as a simple PM. The spectrum of PVP-K30 exhibited a prominent peak at 1658 cm^−1^, corresponding to C=O symmetric stretching of the carbonyl group. Additional peaks were observed at 1420 cm^−1^ (CH_2_ bending), 1371 cm^−1^ (O–H in-plane bending), and 1282 cm^−1^ (C–H deformation) [32].

**Figure 12:**
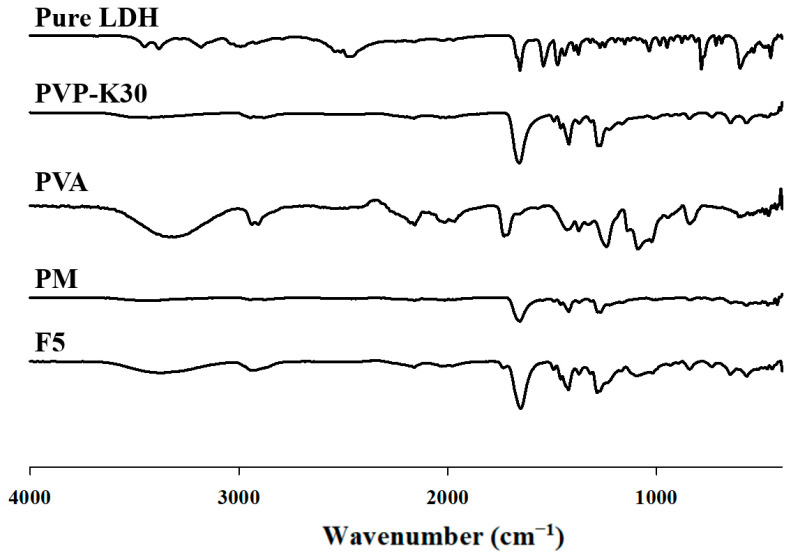
FTIR spectra.

As shown in Figure 12, the FTIR spectrum of PVA exhibited a broad peak at 3304 cm^−1^, corresponding to O–H stretching vibrations and confirming the presence of hydroxyl groups. Peaks at 2907 and 2938 cm^−1^ were assigned to asymmetric and symmetric CH_2_ stretching vibrations, respectively. Further peaks at 1429 and 1374 cm^−1^ corresponded to a combination of O–H in-plane bending and C–H shaking, while the peak at 1080 cm^−1^ was associated with C–O stretching. The ATR-FTIR spectrum of the PVA/PVP blend resembled those of the individual polymers but displayed notable differences. Specifically, the O–H stretching band shifted to 3431 cm^−1^, and changes in absorbance were observed within the ranges of 1800 to 1500 cm^−1^ and 1260 to 1000 cm^−1^. Furthermore, the C=O stretching band of PVP significantly shifted from 1658 cm^−1^ to 1649–1655 cm^−1^. These changes indicate the formation of intermolecular hydrogen bonds between the hydroxyl groups of PVA and the carbonyl groups of PVP, providing strong evidence of successful cross-linking and interaction between PVA and PVP [34].

### 3.5. Drug Content of MNs

The lidocaine hydrochloride content in the MN formulations was analyzed to evaluate formulation accuracy. As shown in Table 3, formulations F4, F5, and F6 exhibited drug content recovery rates between 95% and 105% of the theoretical values, confirming high formulation accuracy and uniform drug distribution.

### 3.6. In Vitro Release Results

**Figure 13:**
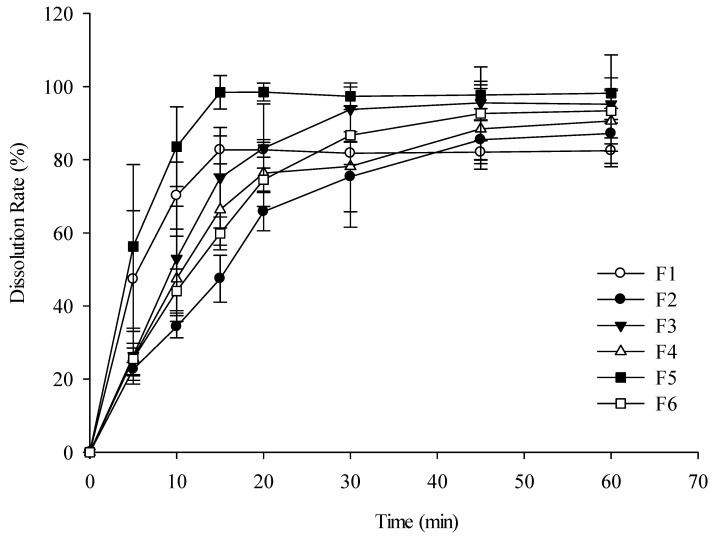
Dissolution profile of MNs in pH 7.4 buffer. Each value represents the mean ± SD (*n* = 3).

The in vitro release profiles of the PVP- and PVA-based MN formulations were evaluated in PBS (pH 7.4) (Figure 13). The PVP-only formulation (F1) exhibited rapid dissolution, whereas the PVA-only formulation (F2) showed a significantly slower dissolution rate. This difference is attributed to the semi-crystalline structure of PVA, which acts as a drug diffusion barrier compared with the higher aqueous solubility of PVP. Consequently, formulation F6, with a higher PVA content, demonstrated a slower drug release rate than the other PVP–PVA blends.

The enhanced dissolution observed in the PVP–PVA blends can be explained by the complementary roles of the two polymers; PVP’s high aqueous solubility promotes rapid hydration and drug diffusion, while PVA provides structural integrity that prevents premature matrix collapse; together, this synergy yields faster and more consistent dissolution than pure PVA systems [35].

Furthermore, formulations with higher PVP content positively correlated with faster drug release. F2 and F5 achieved over 70% cumulative drug release within 10 min; however, only F5 was selected for further study, as F2 suffered from poor mechanical strength due to its PVP-only composition.

### 3.7. Ex Vivo Skin Permeation Results

The cumulative release profile of lidocaine from MNs was evaluated using ex vivo permeation tests with pig skin and analyzed in a Franz diffusion cell system. In the preliminary insertion and penetration depth studies, rat skin was used; however, because it is considerably thinner and more elastic than human skin, direct extrapolation of penetration depth may lead to overestimation. To provide more clinically relevant insights, pig skin was chosen for the ex vivo permeation experiments, as its thickness and barrier properties more closely resemble those of human skin. As a comparative control, EMLA cream—a commercially available local anesthetic widely used in clinical practice—was included. According to the manufacturer’s instructions, EMLA requires at least 60 min of application to achieve adequate anesthesia [36], with therapeutic concentrations for pain relief reported at approximately 100 ng/mg [37].

**Figure 14:**
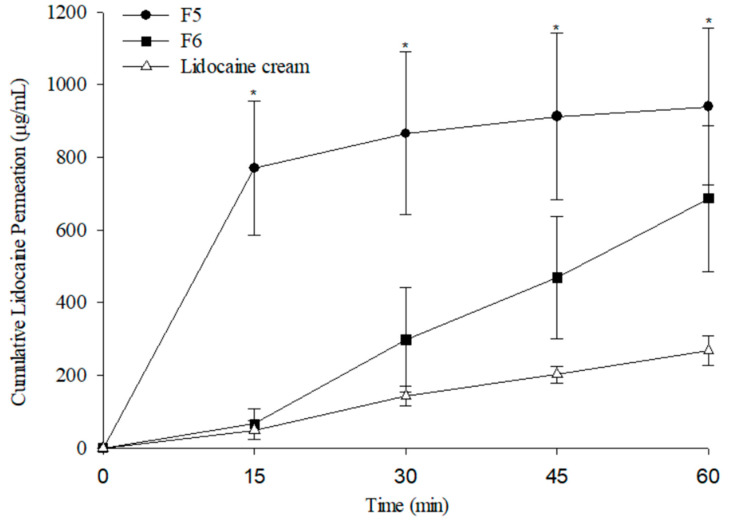
Skin permeability of MNs as a function of lidocaine amount. Each value is present as (mean ± SD, *n* = 3) (* *p* < 0.05).

As shown in Figure 14, formulation F5 demonstrated a significantly higher lidocaine permeation rate than EMLA cream (*p* < 0.05). This result is consistent with the in vitro dissolution results, where F5, due to its higher PVP content, exhibited a faster dissolution rate than other PVP/PVA blends. Polymers with faster dissolution rates release drugs more rapidly, enhancing percutaneous permeation. Consequently, F5 achieved lidocaine permeation levels exceeding 600 µg/mL within 15 min, approximately three times greater than the 200 µg/mL delivered by EMLA cream after 60 min. In contrast, formulation F6 reached therapeutic concentrations approximately 30 min post-application.

The enhanced transdermal delivery efficiency of the MN formulations can be attributed to their ability to penetrate the stratum corneum and create microchannels, thereby facilitating rapid drug transport into the skin. Overall, MN-based formulations achieved superior permeation profiles compared with conventional lidocaine cream. These findings suggest that MN systems represent a promising alternative for rapid drug release and adequate local anesthesia, potentially reducing anesthetic onset time and minimizing patient discomfort during clinical procedures.

## 4. Conclusions

In this study, dissolvable MNs were successfully developed using the biocompatible polymers PVP and PVA to achieve rapid and efficient transdermal delivery of lidocaine. The influence of PVP and PVA concentrations on morphological characteristics, thermal behavior (DSC), crystal structure (XRD), chemical interactions (FTIR), mechanical strength, and in vitro dissolution and skin permeation profiles was thoroughly evaluated. The MNs exhibited a uniform conical geometry with sharp tips, ensuring effective skin insertion. Among the tested formulations, the optimized PVP/PVA ratio of 20:5 demonstrated superior mechanical strength and rapid drug release in vitro. These findings demonstrate the potential of this MN system as an effective transdermal drug delivery platform with enhanced skin permeability compared to conventional topical formulations. Overall, the results suggest that this approach may enable the development of practical, fast-acting, and patient-friendly transdermal therapeutic systems.

## Figures and Tables

**Table 1:** Formulation of lidocaine–PVA/PVP MNs.

Formulation	Concentration (*w*/*w*)			
	API (%)	PVP (%)	PVA (%)	DW (%)
F1	5	30	-	65
F2	5	-	20	75
F3	5	10	10	75
F4	5	15	15	65
F5	5	20	5	70
F6	5	5	20	70

**Table 2:** Size reduction and mechanical strength test of DMNs (mean ± SD, *n* = 3).

	Before Test (µm)	After Test (µm)	Size Reduction (%)
F1	580.33 ± 59.34	305 ± 30.18	47.44 ± 1.83
F2	522.22 ± 33.33	311.44 ± 20.4	40.36 ± 4.03
F3	595.67 ± 5.05	404.67 ± 9.21	32.06 ±1.55
F4	616.22 ± 11.7	434.22 ± 11.76	29.53 ± 1.28
F5	636.22 ± 9.34	454.33 ± 5.29	28.59 ± 1.17
F6	534.67 ± 18.82	417 ± 33.41	22.01 ± 4.49

**Table 3:** Drug content of MNs.

Formulation	Drug Content (%)
F1	87.94 ± 2.61
F2	86.69 ± 1.36
F3	92.13 ± 3.8
F4	96.05 ± 7.85
F5	98.28 ± 4.92
F6	98.18 ± 3.8

## Data Availability

The original contributions presented in the study are included in the article. Further inquiries can be directed to the corresponding authors.
